# Quantum interference features and thermoelectric properties of macrocyclic-single molecules: theoretical and modelling investigation†

**DOI:** 10.1039/d4na00541d

**Published:** 2024-10-02

**Authors:** Sarah Hussein Halboos, Oday A. Al-Owaedi, Enas M. Al-Robayi

**Affiliations:** a Department of Laser Physics, College of Science for Women, University of Babylon Hilla 51001 Iraq oday.alowaedi@uobabylon.edu.iq; b Al-Zahrawi University College, Karbala Najaf-Karbala Street 56001 Iraq

## Abstract

The quantum interference effect on the thermoelectric properties of cycloparaphenylacetylene-based molecular junctions was investigated theoretically using a combination of density functional theory (DFT) methods, a tight binding (Hückel) model (TBHM) and quantum transport theory (QTT). Manipulating the unique conjugation function of these molecules not only creates a quantum interference (QI) but it is also a robust strategy for improving the thermoelectric properties of these molecules. QI controls the transport behaviour and decreases the electrical conductance (*G*) from 0.14 × 10^−7^ to 0.67 × 10^−11^ S, as well as enhancing the Seebeck coefficient (*S*) from 14.4 to 294 μV K^−1^, and promoting the electronic figure of merit (*Z*_el_*T*) from 0.008 to 1.8, making these molecules promising candidates for thermoelectric applications.

## Abstract 2

Cycloparaphenylene molecules not only represent a promising host to examine quantum phenomena but also offer distinct electric and thermoelectric properties to explore new designs and features of thermoelectric materials.

## Graphical abstract

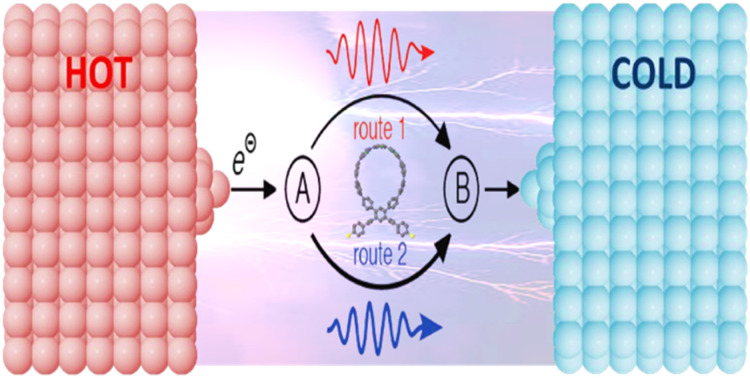

## Introduction

The tunneling transport process across the source|molecule|drain has attracted wide interest and is depicted as a coherent transport.^1^ Quantum interference (QI)^2^ is one of the most important phenomena that control^3,4^ the tunneling transport, and consequently impacts the properties of single-molecule junctions.^5–12^ The attractive functionality of π-conjugated molecules^13^ such as cycloparaphenylene (CPP)^14^ compounds makes them a target of a wide range of investigations.^15–21^ The efforts to form carbon nanotubes with precise structure have led to the development of CPP compounds, and these macrocyclic structures are composed of *para*-linked phenylene rings.^22–26^ Lambert *et al.*^27^ have demonstrated that cycloparaphenylene (CPP) macrocycles show a high electrical conductance due to the topological nature of the de Broglie wave created by electrons injected into the macrocycle from the source. The potential applications of CPP and its derivatives range from organic electronics^28,29^ to supramolecular sensing^30–32^ and bioimaging.^33^ Cycloparaphenylene and its derivatives could be perfect for exploring quantum interference (QI), since they provide a powerful strategy to investigate the propagation of de Broglie waves through the source|molecule|drain configuration. Oday A. Al-Owaedi^34^ proved that the multiple molecular templates of cycloparaphenylene molecules are an ideal host to inspect the quantum interference, and demonstrated that the destructive quantum interference (DQI) influenced the thermoelectric properties of these molecules and raised the Seebeck coefficient (*S*) from 3.13 to 37.24 μV K^−1^.

In addition, there are many interesting phenomena have been found in cycloparaphenylene-based devices, such as a negative differential resistance (NDR),^35^ highly nonlinear *I*–*V* relationship,^36^ electrical switching^37,38^*etc.* Because of its wide applications, *e.g.*, logic, memory, and amplification, NDR has attracted a lot of attention. The spectroscopic^15–18^ and optical^19–21^ properties of CPP molecules have been studied widely. Bryan M. Wong *et al.*^39^ found that the first electronic excitation in chiral nanorings is allowed because of a strong geometric symmetry breaking, which proves that cycloparaphenylene molecules possess extremely interesting optoelectronic properties with excitation energies increasing as a function of size, which is in contrast to typical quantum confinement effects. In this context, manipulating the transport paths of de Broglie waves through this kind of molecule will undoubtedly lead to the emergence of the quantum interference phenomenon.^40–49^ Herein, we explore the influence of QI on the electronic, thermoelectric and spectroscopic properties of CPP-based molecular junctions using a combination of density functional theory (DFT) methods,^50,51^ a tight binding (Hückel) model (TBHM)^52^ and quantum transport theory (QTT).^53–62^

## Computational methods

The initial optimization of gas phase molecules and isosurfaces calculations were carried out at the B3LYP level of theory^63^ with the 6-31G** basis set^64,65^ using density functional theory (DFT) and time-dependent DFT (TD-DFT)^66^ respectively. The geometrical optimization of all gold|CPP|gold configurations under investigation in this work was accomplished by the implementation of DFT^67,68^ in the SIESTA^67^ code, as shown in Fig. 4 and S2 (see the ESI†). The generalized gradient approximation (GGA) of the exchange and correlation functional is used with a double-ζ polarized (DZP) basis set, a real-space grid defined with an equivalent energy cut-off of 250 Ry. The geometry optimization for each structure is performed for forces smaller than 20 meV Å^−1^. The mean-field Hamiltonian obtained from the converged DFT calculations was combined with Gollum^53^ code. Quantum transport theory (QTT)^54,69^ implemented in Gollum has been used to calculate the electronic and thermoelectric properties of all molecular junctions. The optimized molecules have been attached to two (111)-directed pyramidal gold electrodes. Each electrode was constructed of seven layers of (111)-oriented bulk gold, with each layer consisting of 6 × 6 atoms and a layer spacing of 0.235 nm, which were used to create the molecular junctions. These layers were then further repeated to yield infinitely long gold electrodes carrying current, as shown in Fig. 4 (see the ESI for more details†).

## Results and discussion

**Fig. 1:**
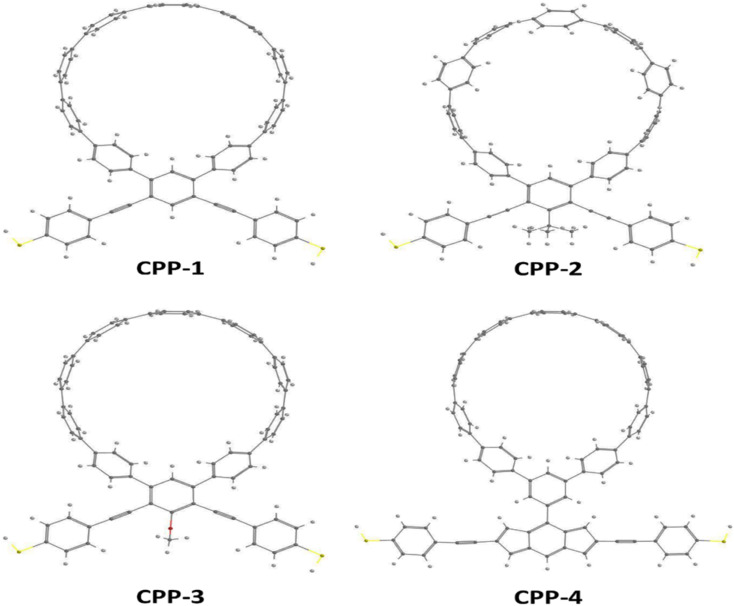
Schematic illustration of CPP molecules. CPP-1, CPP-2 and CPP-3 molecules consist of OPE with methylpropane and methoxy pendant groups for CPP-2 and CPP-3 respectively. The CPP-4 molecule has an indacene structure. White balls are hydrogen atoms, gray balls are carbon atoms, yellow balls are sulfur atoms and the red ball is an oxygen atom.

Fig. 1 shows that each molecule under investigation in this work is a necklace consisting of a mixture of two molecules. The first three molecules CPP-1, CPP-2 and CPP-3 consist of an oligo(phenylene ethylene) (OPE) molecule incorporated with a cycloparaphenylene (CPP) molecule, while molecule CPP-4 is a necklace consisting of a combination of a CPP molecule plus an indacene structure. All molecules are terminated by a thiol compound as an anchor group. In addition, molecules CPP-1, CPP-2 and CPP-3 possess *meta*-connectivity, while a connection of *para* is the distinguishing feature of molecule CPP-4. Furthermore, 2-methylpropane is the pendant structure of the molecule CPP-2, while the methoxy group represents the pendant group of the molecule CPP-3, and molecule CPP-4 contains an indacene compound as a pendant group.

**Fig. 2:**
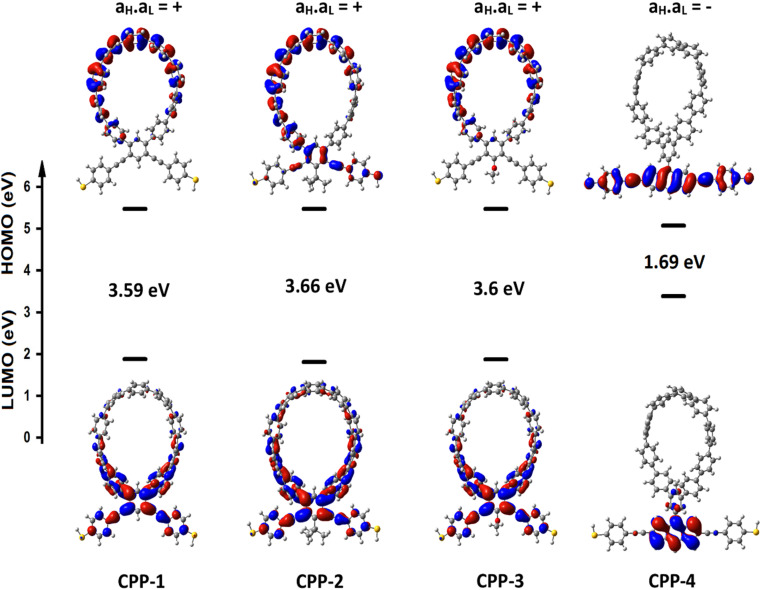
The highest occupied and lowest unoccupied molecular orbitals (HOMOs and LUMOs) (isosurfaces ±0.02 (e bohr^−3^)^1/2^), the blue part is a positive sign, and the red part is a negative sign. *a*_H_·*a*_L_ is the multiplication of the HOMO and LUMO amplitudes. As an example, the HOMO and LUMO for the CPP-4 molecule possess different signs, so the multiplication of molecular orbital amplitudes (*a*_H_·*a*_L_) is a negative sign and the molecule exhibits a constructive quantum interference (CQI).

Obviously, the HOMOs of the first three molecules (CPP-1, CPP-2 and CPP-4) are localized on the circular part of each molecule, while they extend along the indacene structure for the fourth molecule (CPP-4). In contrast, LUMOs have a significant weight on the OPE part of the molecule for CPP-1, CPP-2 and CPP-3, while they have less weight on the indacene compound for the molecule CPP-4. In addition, the HOMOs and LUMO do not have a considerable weight on the circular part of CPP-4. The narrowest HOMO–LUMO gap (1.69 eV) is presented by the fourth molecule (CPP-4). To some extent, the other molecules exhibit gaps of similar values, as shown in Fig. 2 and Table 1.

**Table 1:** Highest occupied molecular orbital (HOMO); lowest unoccupied molecular orbital (LUMO); HOMO–LUMO gap (H–L gap); *A* is the absorption intensity; ^A^*λ*_max_ is the maximum absorption wavelength; *E* is the emission intensity; ^E^*λ*_max_ is the maximum emission wavelength; *f*_em_ is the emission oscillator strength; SS is the Stokes shift

Molecule	HOMO (eV)	LUMO (eV)	H–L gap (eV)	*A* (a.u.)	^A^ *λ*_Max_ (nm)	*E* (a.u.)	^E^ *λ*_Max_ (nm)	*f*_em_	SS (nm)
CPP-1	5.47	1.88	3.59	1614.6	352	62 532	375	1.34	23
CPP-2	5.47	1.81	3.66	1290.6	348	49 387.4	372	0.95	24
CPP-3	5.47	1.87	3.6	1931.7	350	71 303.1	374	1.3	24
CPP-4	5.07	3.38	1.69	24.25	536	1871.2	592	0.04	56

In order to explore the impact of connectivity type and to prove the existence of QI^66^ in CPP molecules, as part of the current investigation an orbital analysis was performed, and it demonstrated that the destructive quantum interference (DQI) dominates the transport of most molecular junctions, as shown in Fig. 2. Lambert *et al.*^70^ have reported an orbital symmetry rule. Magic ratio theory^67^ is based on utilising the exact core Green's function, defined by:

1 *g*(*E*) = (IE − *H*)^−1^

In the literature, various approximations to *g*(*E*) are discussed, one of which involves the approximation of including only the contributions to *g*(*E*) from the HOMO and LUMO. If the amplitudes of the HOMO on sites a and b are denoted 
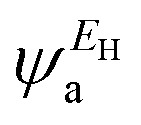
 and 
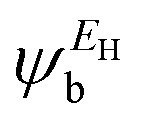
 and the amplitudes of the LUMO are 
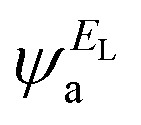
 and 
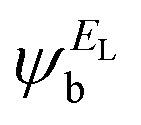
, then if the contributions from all other orbitals are ignored, then, a crude approximation to Green's function *g*_ba_(*E*) is2
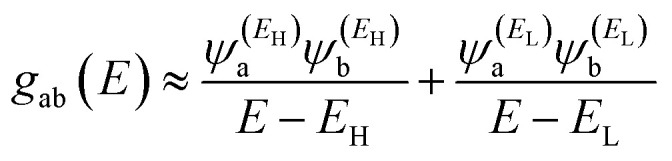
where *E*_H_ and *E*_L_ are the energies of the HOMO and LUMO respectively. If the HOMO product 
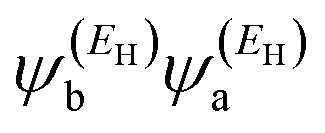
 has the same sign as the LUMO product 
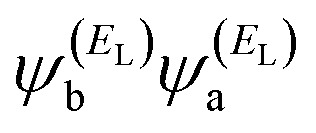
 then the right-hand side of eqn (2) will vanish at some energy *E* in the range *E*_H_ ≤ *E* ≤ *E*_L_. That is for some energy *E* within the HOMO–LUMO gap. In this case, one can say that the HOMO and the LUMO interfere destructively. On the other hand, if the HOMO and LUMO products have opposite signs then the right hand side of eqn (2) will not vanish within the HOMO–LUMO gap and one can say that the HOMO and LUMO interfere constructively within the gap (they could of course interfere destructively at some other energy *E* outside the gap). When the right-hand side of eqn (2) vanishes, the main contribution to *g*_ba_(*E*) comes from all other orbitals, so in general eqn (2) could be a poor approximation. One exception to this occurs when the lattice is bipartite, because the Coulson–Rushbrooke (CR) theorem^68^ tells us that if a and b are both even or both odd, then the orbital products on opposite sides of eqn (3) and (4) have the same sign. Consequently, the HOMO and LUMO interfere destructively, while all other pairs of orbitals interfere destructively, leading to trivial zeros in the magic number table,^70^ for which *g*_ba_(0) = 0.3
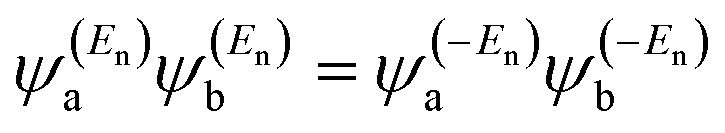
4
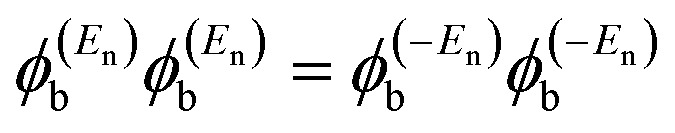
where ±*E*_n_ are eigenvalues that come in ± pairs and the eigenstate belonging to −*E*_n_ is related to the eigenstate belonging to *E*_n_.

Obviously, this exact cancellation is a property of bipartite lattices only, but based on its success for bipartite lattices, one might suppose that eqn (2) is a reasonable approximation, for other lattices. Nevertheless, as pointed out by Yoshizawa *et al.*,^71–74^ since orbitals such as those in Fig. 2 are often available from DFT calculations, it can be helpful to examine the question of whether or not the HOMO and LUMO (or indeed any other pair of orbitals) interfere destructively or constructively, by examining the colours of orbitals. This is simplified by writing eqn (2) in the form5
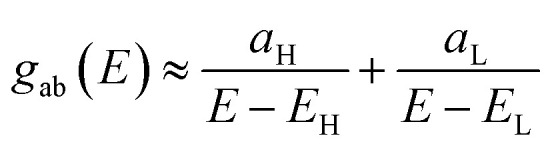
where 
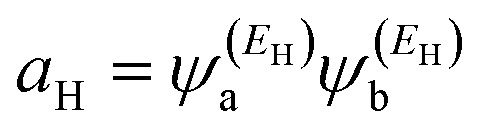
 and 
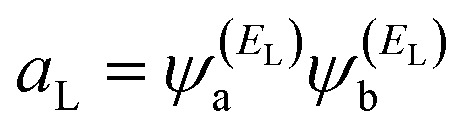
. If the HOMO product *a*_H_ has the same sign as the LUMO product *a*_L_ then the right-hand side of eqn (5) will vanish for some energy *E* in the range *E*_H_ ≤ *E* ≤ *E*_L_. In other words, the HOMO and LUMO will interfere destructively at some energy within the HOMO–LUMO gap. However, this does not mean that the exact *g*_ba_(*E*) will vanish. Indeed, if the right hand side of (5) vanishes, then the contributions from all other orbitals become the dominant terms.^75^ Nevertheless, this is an appealing method of identifying QI effects in molecules and describing their qualitative features.^76^

**Fig. 3:**
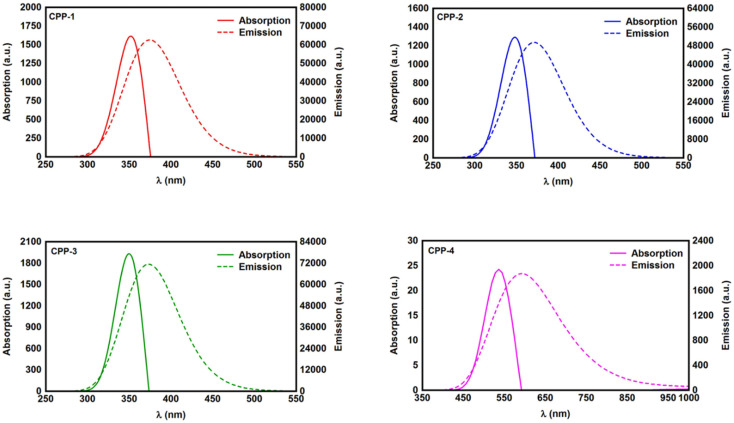
UV/Vis absorption spectra (solid curves) and emission spectra (dashed curves) for all molecules.

Studying the spectroscopic properties has become one of the necessary factors to complete an integrated investigation for any system in order to predicate the appropriate applications for that system. Therefore, the absorption and emission spectra of CPP molecules are topics of great interest for many investigations.^15–21^Fig. 3 and Table 1 show that the UV/visible absorption and emission spectra of the first three molecules (CPP-1, CPP-2 and CPP-4) exhibit a blueshift, since the range of the absorption spectrum extends from 350 to 352 nm, and the emission spectrum ranges from 372 to 375 nm. These results are consistent with the literature.^15–21^ On the other hand, molecule CPP-4 shows a visible light region, since its absorption and emission spectra are 536 and 592 nm respectively. Table 1 shows one of the most important parameters in optoelectronics applications, which is the emission oscillator strength (*f*_em_).^77^ Theoretically, for a given PL material, *f*_em_ is directly proportional to the emission cross section (*σ*_em_) and it is given by:^78^6
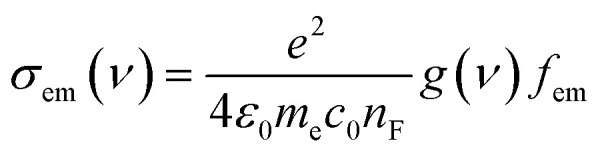
where *e* is the electron charge, *ε*_0_ is the vacuum permittivity, *m*_e_ is the mass of an electron, *c*_0_ is the speed of light, *n*_F_ is the refractive index of the gain material, *ν* is the frequency of the corresponding emission, and g(*ν*) is the normalized line shape function with 
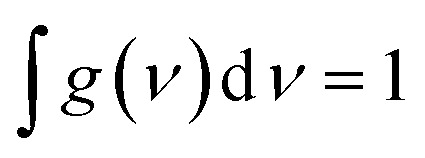
. Interestingly, the emission oscillator strengths (*f*_em_) of CPP-1, CPP-2 and CPP-3 are 1.34, 0.95 and 1.3 respectively, whereas molecule CPP-4 shows the lowest value of *f*_em_ (0.04), as shown in Table 1. These results suggest that cycloparaphenylene-single molecules are promising candidates for optoelectronics applications.

**Fig. 4:**
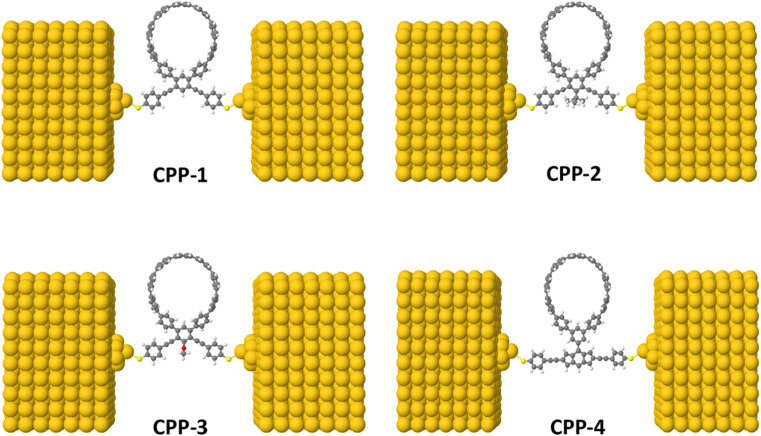
Theoretical models of optimized molecular junctions.

In this work the transmission coefficient *T*(*E*) has been calculated by attaching the optimized molecules with two (111)-directed gold electrodes, as shown in Fig. 4. From these molecular junctions the electronic and thermoelectric properties were calculated using Gollum code.^48,79^ The transmission coefficient according to Landauer–Büttiker^80^ formalism is given by:

7 *T*(*E*) = *T*_r_{*Γ*_R_(*E*)*G*^R^(*E*)*Γ*_L_(*E*)*G*^R†^(*E*)}

where8
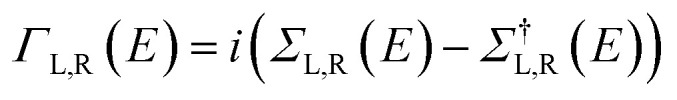
where *Γ*_L,R_ describes the level broadening due to the coupling between the left (L) and right (R) electrodes and the central scattering region, and *Σ*_L,R_(*E*) is the retarded self-energies associated with this coupling.

9 *G*^R^ = (*EX* − *H* − *Σ*_L_ − *Σ*_R_)^−1^

where *G*^R^ is the retarded Green's function, *H* is the Hamiltonian and *X* is the overlap matrix. The transport properties are then calculated using the Landauer formula:10
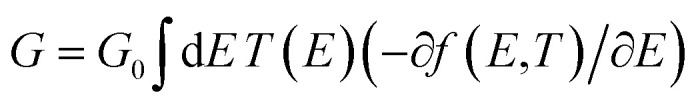
where *G*_0_ = 2*e*^2^/*h* is the conductance quantum, *f*(*E*) = (1 + exp((*E* − *E*_F_)/*k*_B_*T*))^−1^ is the Fermi–Dirac distribution function, *T* is the temperature and *k*_B_ = 8.6 × 10^−5^ eV K^−1^ is Boltzmann's constant. Regarding the relaxed geometries shown in Fig. 4, it is obvious that all molecules were accommodated appropriately between electrodes, with an angle of 52° between the anchor group (thiolate (RS^−^)) and gold. These results are in excellent agreement with the literature.^81,82^

**Fig. 5:**
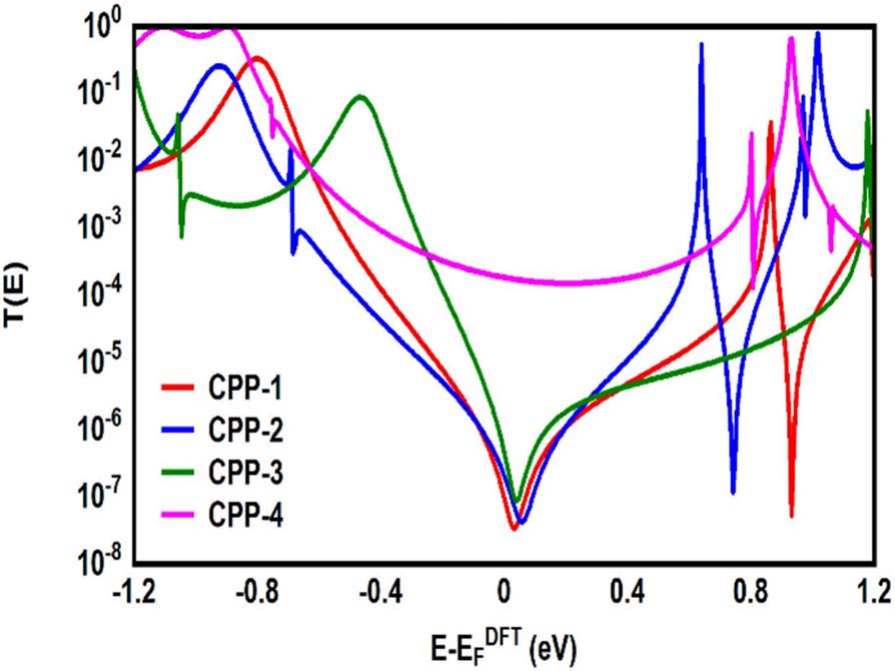
Transmission coefficient *T*(*E*) as a function of electron energy for all molecules.

Many investigations^83,84^ have demonstrated that quantum interference (QI) arises from the tunneling of electrons through various molecular orbitals with different phases. Both of interfering and noninterfering effects are comprised in the transmission function, so the single interference between molecular orbitals cannot be diagnosed from the transmission function directly. Latha Venkataraman *et al.*^84^ have deconstructed the interferences of the molecular orbitals and established a powerful method to arrange these interferences in a matrix and display them pictorially as a QI map, which allows one to easily identify individual QI effects. The current study predicates that the HOMO–LUMO orbitals interfered destructively at the middle of the HOMO–LUMO gap, near the theoretical Fermi energy (0.0 eV) with transmission functions featuring antiresonances, as shown in Fig. 5. These results are in excellent agreement with various pen-and-paper methods^85–87^ that predicted the existence of antiresonances at the Fermi energy. The origin of the destructive quantum interference (DQI) is the node at the *meta* position in the HOMO which prevents electronic coupling between two *meta* substituents. Therefore, *meta*-linked wires show poor conductance and DQI for the same reason that electrophilic aromatic substitution reactions with electron-rich substituents, in general, do not occur at the *meta*-position.^88,89^

Fig. 5 shows the transmission coefficient *T*(*E*)^90,91^ of source|molecule|drain junctions. The transmission coefficient curves of the first three molecules (CPP-1, CPP-2 and CPP-3) show robust antiresonance features as a representation of the destructive quantum interference (DQI). These features are located at the middle of the HOMO–LUMO gap, toward the HOMO peak. In contrast, the antiresonance features disappeared in the transmission curve of the CPP-4 molecule, as well as presenting the highest value of *T*(*E*) along a wide range of energies, which refers to the constructive quantum interference (CQI). The order of *T*(*E*) is CPP-4 > CPP-3 > CPP-2 > CPP-1, as shown in Table 2. In addition, the theoretical Fermi energy (0.0 eV) lies within the HOMO–LUMO gap towards the HOMO resonance, which indicates the HOMO-dominated transport mechanism.^92,93^Fig. 5 reflects the fact that the destructive quantum interference (DQI) dominates the transport process through the first three molecules, and increases the values of Seebeck coefficient (*S*) and the electronic figure of merit (*Z*_el_*T*), and the transport behavior varies from one molecule to another, and this difference in turn will lead to a difference in the slope of the transmission coefficient *T*(*E*), which affects the values of the Seebeck coefficient.

**Table 2:** Transmission coefficient *T*(*E*); the number of electrons transferred from the molecule to electrodes (*Γ*); HOMO–LUMO gap (*Ω*^a^) of CPP-based molecular junctions; HOMO–LUMO gap (*Ω*^b^) of OPE and indacene-based molecular junctions

Molecule	*T*(*E*)	*Γ*	*Ω* ^a^ (eV)	*Ω* ^b^ (eV)
CPP-1	8.64 × 10^−8^	0.73	2.085	2.37
CPP-2	1.82 × 10^−7^	0.8	1.937	2.24
CPP-3	4.02 × 10^−7^	0.85	2.07	2.6
CPP-4	1.8 × 10^−4^	2.3	1.8	2.25

**Fig. 6:**
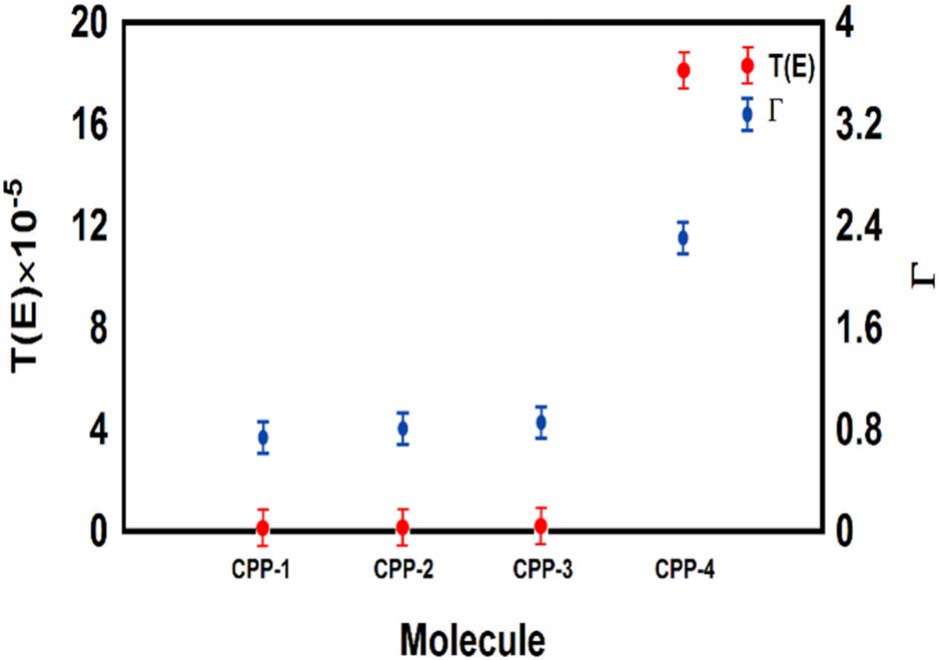
The number of electrons transferred from the molecule to electrodes (*Γ*) against the transmission coefficient *T*(*E*) for all molecules.

The *meta*-linked structures have an extremely small number of transferred electrons (*Γ*), which leads to an impalpable *T*(*E*). In contrast, molecule CPP-4 with *para* connection exhibits a high value of *T*(*E*) due to the constructive quantum interference (CQI) (Fig. 6).

The molecule length of all molecules is consistent with a dominant contribution from the coherent tunneling mechanism.^94–98^ The rectangular tunnel barrier model^99^ states that the electrical conductance through a single molecule (barrier) decreases exponentially with the length of the barrier, according to eqn (11).

11 *T*(*E*) ∝ e^−*βl*^

where *T*(*E*) is the transmission coefficient, *β* is the electronic decay constant and *l* is the tunnelling distance. Herein, the molecule CPP-4 represents the longest molecule, but it shows the highest value of *T*(*E*). In contrast, CPP-1, CPP-2 and CPP-3 molecules possess the same molecule length, which is the shortest length, but they show transmission values that are lower by several fold than that of the CPP-4 molecule, as shown in Table 2 and Fig. 5. Therefore, these results cannot be explained according to Kirchhoff's law; instead, they are evidence of the quantum interference effect.

**Fig. 7:**
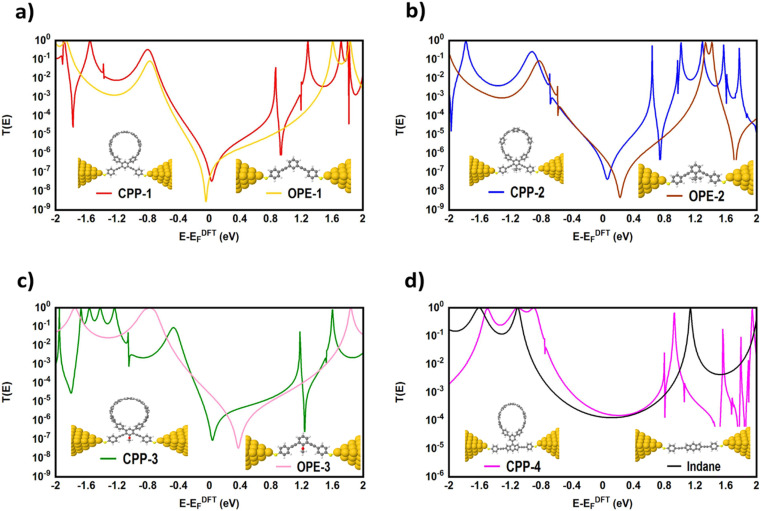
Transmission coefficient *T*(*E*) as a function of electron energy; (a) *T*(*E*) of CPP-1 and OPE-1 molecular junctions; (b) *T*(*E*) of CPP-2 and OPE-2 molecular junctions; (c) *T*(*E*) of CPP-3 and OPE-3 molecular junctions; (d) *T*(*E*) of CPP-4 and indacene molecular junctions.

**Fig. 8:**
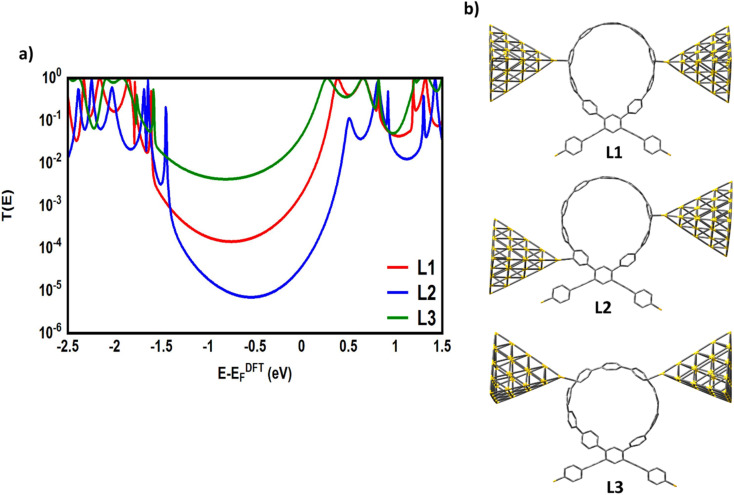
(a) Transmission coefficient *T*(*E*) as a function of electron energy of the CPP-1 molecular junction as an example; (b) theoretical models of optimized molecular junctions in different junction formation probabilities. L1 is the *meta*–*meta* connection; L2 is the *meta*–*ortho* connection; L3 is the *para*–*para* connection.

To gain a deeper understanding of the role of macrocyclic units and their effect on the properties of the molecular junctions under investigation in this work, we have calculated the transmission coefficient *T*(*E*) of molecular junctions that consist of only oligo(phenylene ethylene) (OPE) and indacene molecules and compared the results of both systems (with and without macrocyclic units), as shown in Fig. 7. Obviously, the absence of the macrocyclic unit has led to the widening of the HOMO–LUMO gap, as the value of this gap for molecular junctions that include a macrocyclic unit is smaller (on average) by 0.39 eV than that of the unit-free structures, as shown in Table 2. This result could be ascribed to the quantum size effect.^100^ On the other hand, the presence of the circular units impacts the slope of the transmission coefficient, which undoubtedly affects the Seebeck coefficient characteristics (see Fig. 11a). It is well known that the *meta*-connection leads to a destructive quantum interference (DQI) and an appearance of the antiresonance feature. Herein, the curve of the transmission coefficient of structures with a wheel shows two antiresonances, one at the middle of the HOMO–LUMO gap, near the theoretical Fermi energy (0.0 eV), and the second located between 0.8 and 1.2 eV, while the wheel-free compositions have just one antiresonance feature. Moreover, the CPP-4 molecule possesses a *para* connectivity, and the effect of the constructive quantum interference (CQI) is prominent *via* the highest transmission coefficient value, but there is also a small antiresonance feature in the transmission curve. In contrast, the transmission curve of the structure with the same backbone (indacene) and without a macrocyclic unit is completely free of antiresonance features. Based on these results, it can be concluded that the origin of the quantum interferences (QIs) is the connectivity type (*meta* or *para*) with an important contribution of the macrocyclic units. This contribution may be the result of a tilt or twist in the circular rings during the accommodation between electrodes. Indeed, these results and behaviour need more exploring and investigation. According to the rectangular tunnel barrier model^99^ and eqn (11), *T*(*E*) decreases exponentially with the length of the tunneling distance. The macrocyclic unit-based molecular junctions have the longest distance, but their transmission coefficient is the highest over a wide range of energies, as shown in Fig. 7. This could be explained in terms of the existence of more than one probability for the junction formation. The strong interactions between the extended π-system and gold electrodes create a good chance to form a molecular junction,^27^ and the high binding energy between sulfur (S) atoms and gold (Au) atoms^101^ represents another strong probability for the junction formation and molecule. This investigation took into account both of these probabilities, as shown in Fig. 7 and 8.

The theoretical models shown in Fig. 8 have simulated the probable contacts of gold electrodes to carbon atoms in the circular wheel of structure CPP-1 with various connections. The L1 model involves the formation of the molecular junction *via* a *meta*–*meta* connection. This kind of connectivity is well known to cause a difference in the phase of the traveling electronic waves, which in turn leads to a destructive quantum interference (DQI), that decreases the transmission coefficient as shown in Fig. 8a. In contrast, the L3 model shows the structure of the molecular junction with a *para*–*para* connection. This system shows the highest *T*(*E*) due to a constructive quantum interference (CQI). These results could be explained in terms of Mach–Zehnder interferometers.^102^ The de Broglie waves cross identical pathways of L3, which results in wave interference at the same phase, then CQI (*T*(*E*) = |e^*ik*3^ + e^*ik*3^|^2^ = |e|^*ik*3^ = |1 + 1|^2^ = 4), raising the *T*(*E*) of the whole system. In contrast, the different pathways of the L1 model lead to a low *T*(*E*), which could be attributed to DQI (*T*(*E*) = |e^*ik*4^ + e^*ik*2^|^2^ = |e^*i*2π^ + e^*i*π^| = |1 − 1|^2^ = 0). Model L2 exhibits a mixed connection consisting of *meta* and *ortho* positions. Interestingly, the transmission value of this structure is mediated between the lowest and highest values of *T*(*E*) of other systems, which could be understood by releasing a predication that the transmission coefficient of this structure is a result of the contributions of constructive (*ortho*) and destructive (*meta*) quantum interferences at the same time. These results open an important window to design new materials to control and utilize the quantum interference in different electronic applications. On the other hand, it could be observed that all *T*(*E*) curves are completely clear of any mark of the antiresonance feature, and so there is no dramatic difference between the slope of *T*(*E*) curves. Consequently, it is expected that the Seebeck coefficient of these models will not show promising results, as shown in Fig. S4 (see the ESI†).

**Fig. 9:**
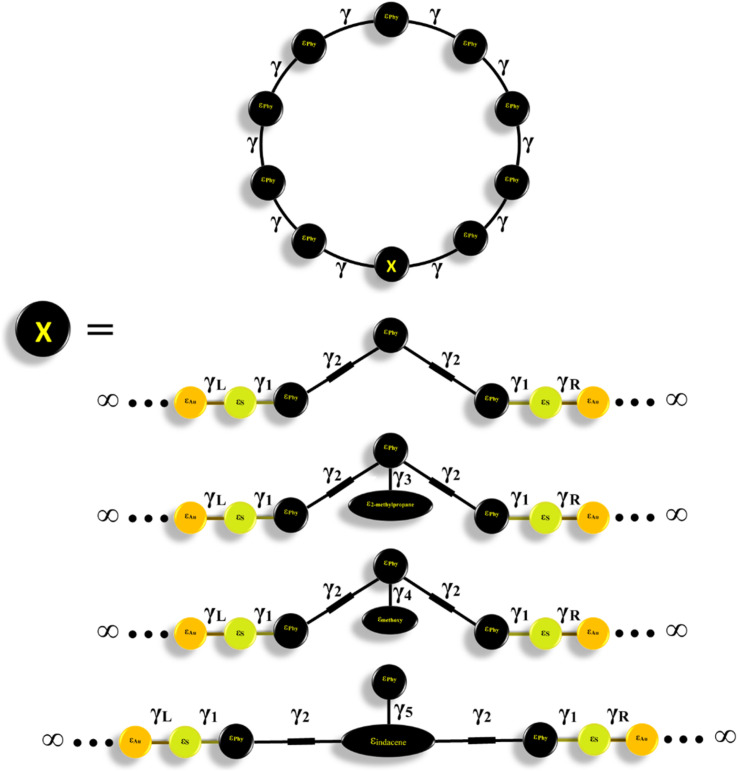
A minimal tight-binding (Hückel) representation of CPP molecules with different intramolecular coupling elements, *γ*, and different onsite energies *ε*.

In seeking to confirm the quantum interference effect and understand the transport behaviour of molecules and the relative effects of different pendant groups, a minimal tight-binding (Hückel) model (TBHM) has been constructed, as shown in Fig. 9. The simplest tight-binding Hamiltonian of the parents is obtained by assigning a site energy *ε* to each diagonal and a nearest neighbor hopping integral *γ* between neighbouring sites, *i.e.*, *H*_*ii*_ = *ε* and *H*_*ij*_ = *γ* if *i*, *j* are the nearest neighbours.

Fig. 9 shows a system connected to two one-dimension electrodes on both sides by weak nearest neighbor coupling of *γ*_R_ and *γ*_L_. The tight-binding (Hückel) model (TBHM) neglects the interactions between electrons, which is considered a major defect, but it remains one of the widely used methods to visualize and understand the electronic properties of molecular junctions.^103^ In addition, the energy levels produced by this kind of computational method are diminished by a few electron voltages in comparison with the accurate values relative to those in a vacuum. However, the energy differences are appropriate to compare with DFT calculations and consequently this method is considered a powerful tool to obtain reasonable and precise results that could yield the fundamental physics and resolve the problems.^104^

**Fig. 10:**
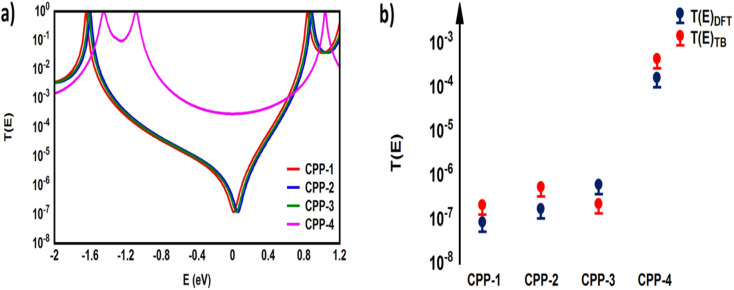
(a) Transmission coefficient as a function of electron energy for all models presented in Fig. 9. The coupling integrals *γ* = *γ*_1_ = *γ*_L_ = *γ*_R_ = 0.5 eV, *γ*_2_ = 0.3 eV, *γ*_3_ = 2.2, *γ*_4_ = 0.5, and *γ*_5_ = 0.9 eV are fixed for all models. The on-site energies are *ε*_phy_ = 0.5 eV for CPP-1, *ε*_2-methylpropane_ = 0.7 eV for CPP-2, *ε*_methoxy_ = 0.7 eV for CPP-3 and *ε*_indacene_ = 1.1 eV for CPP-4; (b) representation of the agreement between density functional theory (DFT) and tight-binding (TB) calculations.

Furthermore, TBHM not only takes into account all morphological aspects of molecular junctions, but also assumes that the electron transport is elastic and coherent.^104^Fig. 10 shows the transmission coefficient produced by TBHM as a function of electron energy of all models. If all sites are treated as the same and the on-site energies set to 0 eV and coupling integrals to −1 eV, the transmission values disagree with the DFT results (see the ESI†). However, by adjusting the coupling integrals (*γ* = *γ*_1_ = *γ*_L_ = *γ*_R_ = 0.5 and *γ*_2_ = 0.3 eV) for all models, as well as *γ*_3_ = 2.2, *γ*_4_ = 0.5 and *γ*_5_ = 0.3 eV, then by changing the on-site energies (*ε*_phy_ = 0.5, *ε*_2-methylpropane_ = 0.7, *ε*_methoxy_ = 0.7 and *ε*_indacene_ = 1.1 eV), the TBHM results are reproduced, as shown in Fig. 10a. The 2-methylpropane structure is joined by carbon–carbon sigma bonds allowing them to rotate about these bonds. In terms of Newman projection,^105^ the free rotation around single bonds results in various conformations. These conformations are classified as staggered and eclipsed conformations. Staggered conformations are the lower energy arrangements, whereas eclipsed conformations are higher in energy than staggered conformations due to bond straining. Herein, the tight-binding (TB) model depicts the molecular rotation by changing the value of *γ*_3_ to obtain the appropriate eclipsed conformation. For *γ*_3_ = 2, the TB model produces a DQI-dominated transport behaviour of model CPP-2. The rotation of the methoxy (OMe) pendant group for the CPP-3 molecule has been pictured as *γ*_4_. An excellent agreement between DFT and TB has been reached by adjusting *γ*_4_ to be 2. The results of TB agree with the results of DFT as shown in Fig. 10a, as well as confirming that there are two parameters controlling and switching the quantum interference from CQI to DQI, which are the connectivity type either *para* or *meta*, and the second parameter is the kind of pendant group.

The slope of *T*(*E*) determines the Seebeck coefficient (*S*) and electronic figure of merit (*Z*_el_*T*), which are given by:12
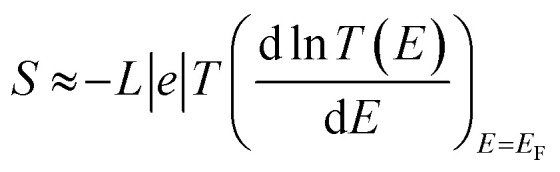
where *L* is the Lorentz number 
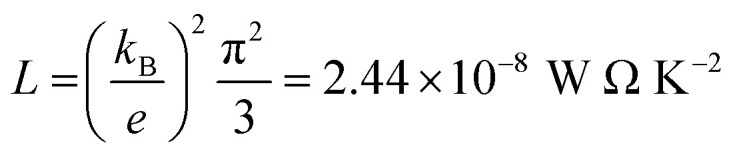
. In other words, *S* is proportional to the negative of the slope of ln *T*(*E*), evaluated at the Fermi energy. Based on the Seebeck coefficient, the power factor was calculated by:

13 *P* = *GS*^2^*T*

where *T* is the temperature (*T* = 300 K), *G* is the electrical conductance and *S* is the Seebeck coefficient. The purely electronic figure of merit (*Z*_el_*T*) is given by:^106,107^14
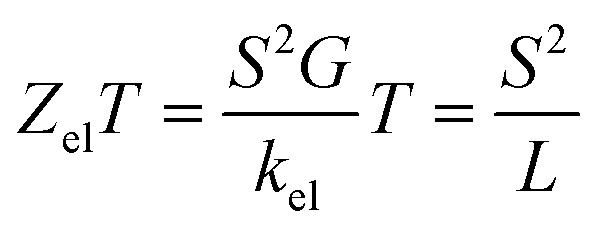
where *k*_el_ is the electron thermal conductance. According to previous studies,^106,107^ the figure of merit in this work has been calculated only based on a purely electronic contribution, as shown in eqn (14).

**Fig. 11:**
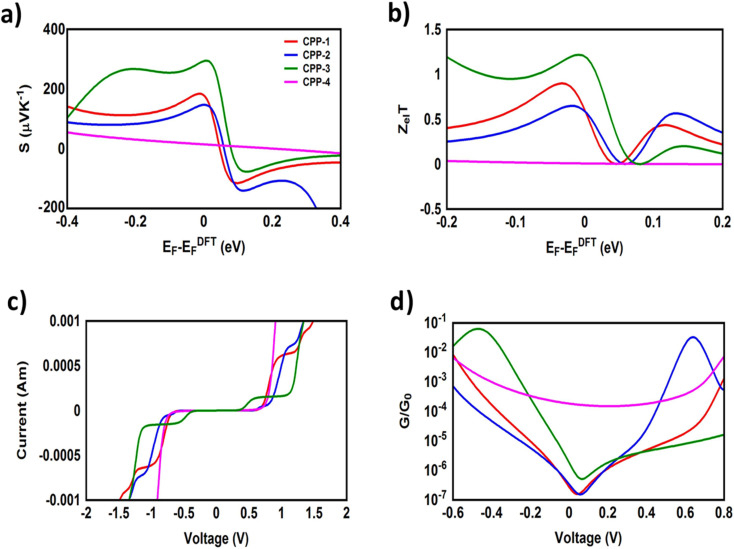
(a) Seebeck coefficient (*S*); (b) electronic figure of merit (*Z*_el_*T*); (c) current–voltage characteristics; and (d) electrical conductance (*G*/*G*_0_) as a function of applied voltage for all molecular junctions.

It is well known that the performance of thermoelectric materials is characterized by an efficient conversion of input heat to electricity.^44,108^ In this context, the enhancement of the power factor (*P*) and electronic figure of merit (*Z*_el_*T*), which depend on the Seebeck coefficient (*S*), is important. Fig. 11a, b and Table 3 show that the highest values of *S* and *Z*_el_*T* (294 μV K^−1^ and 1.12 respectively) have been exhibited by molecule CPP-3. In contrast, molecule CPP-4 presents the lowest values of these parameters (14.4 μV K^−1^ and 0.0081). In addition, molecules CPP-1 and CPP-2 introduce high *S* and *Z*_el_*T* values as shown in Table 3. These results not only established the important role of the existence of the destructive quantum interference in improvement of the *S* and *Z*_el_*T* characteristics but also show a crucial contribution of the pendant groups in promoting these properties. Furthermore, the competition between electrical conductance (*G*) and Seebeck coefficient (*S*) according to eqn (13) led to the power factor order of *P*_CPP-4_ > *P*_CPP-3_ > *P*_CPP-2_ > *P*_CPP-1_. In light of the aforementioned results, these molecules could be considered as promising candidates for thermoelectric applications. The values of transmission coefficient *T*(*E*), Seebeck coefficient (*S*) and electronic figure of merit (*Z*_el_*T*) are found to be higher when the contact Fermi energies are close to the middle of the HOMO–LUMO gap and increase as Fermi energies approach resonance with the highest occupied molecular orbitals (HOMO). To some extent, these results are consistent with an investigation of Milan *et al.*,^109^ since they reported that the electronic and electrical properties depend in a very sensitive manner on the position of the contact Fermi energies within the HOMO–LUMO gap.

**Table 3:** Seebeck coefficient (*S*); electronic figure of merit (*Z*_el_*T*); power factor (*P*); threshold voltage (*V*_th_); and electrical conductance (*G*) for all molecular junctions

Molecule	*S* (μV K^−1^)	*P* (W K^−1^ × 10^−23^)	*Z*_el_ *T*	*V*_th_ (V)	*G* (S)
CPP-1	175	20 518	0.55	0.66	0.67 × 10^−11^
CPP-2	147	30 252.6	0.57	0.64	0.14 × 10^−10^
CPP-3	294	267 951.6	1.12	0.43	0.31 × 10^−10^
CPP-4	14.4	290 304	0.0081	0.69	0.14 × 10^−7^

Fig. 11c, d and Table 3 present the electrical conductance and current–voltage (*I*–*V*) characteristics of all molecular junctions, which are limited to the first and third quadrants of the *I*–*V* plane crossing the origin. Therefore, they are classified as components that consume electric power, and here the importance of the threshold voltage (*V*_th_) value appears. The values of *V*_th_ range from 0.43 to 0.69 V, which makes these molecules promising candidates for electronic applications.

All molecules show a quantum staircase behaviour. Obviously, as the voltage increases, the density of electrons also increases, which leads to an increase in the number of occupied subbands. The dependence of conductance in this case is represented by a set of plateaus separated by steps of height 2*e*^2^/*h*: a stepwise change in the conductance of all molecule channels occurs each time the Fermi level coincides with one of the subbands. Hence, the quantum staircase behaviour could be attributed to the adiabatic transparency of the spin-nondegenerate subbands of these molecules.^110,111^ On the other hand, after the threshold bias voltage, the current increases obviously with the increasing bias voltage. When the bias voltage is further increased to a certain range [2, 2.5] V, the current decreases and the negative differential resistance (NDR) appears, as shown in Fig. 10c and S3.† These results are consistent with the results in the literature.^35^

## Conclusions

Numerous methods and tools have been used to create the destructive quantum interference (DQI) phenomenon. From the current investigation it can be concluded that the cycloparaphenylene-single molecules and their derivatives represent a promising host for creating the quantum phenomena by manipulating the topological properties *via* crucial parameters, which are the connectivity or the pendant group. These parameters not only control the transport behaviour of CPP molecules, but also enhance the thermoelectric properties. Therefore, we believe that these findings will strongly help in developing fast and trustworthy design of molecular electronics and thermoelectric materials.‡‡The results of the current investigation were achieved based on theories and relevant computational methods, which are reported in the ESI.† It contains all details of the theoretical models of all source, molecule and drain configurations. In addition, the ESI† includes the results of the negative differential resistance (NDR) phenomenon for CPP molecules. Furthermore, the calculations and results of the Seebeck coefficient for different connections between the molecule and gold electrodes were mentioned, as well as all details and results of the tight-binding Hückel model (TBHM).^112–115^

## Author contributions

Sarah Hussein Halboosa: investigation, writing – original draft, visualization, calculations. Oday A. Al-Owaedi: supervision, investigation, writing – review & editing, conceptualization, validation, analysis, software, analysis, calculations. Enas M. Al-Robayia: supervision.

## Conflicts of interest

The authors declare no competing financial interest.

## Supplementary Material

NA-006-D4NA00541D-s001

## Data Availability

The data is available in the manuscript and ESI.†
