# Supplementary material for: Quantum interference features and thermoelectric properties of macrocyclic-single molecules: theoretical and modelling investigation
Source: Nanoscale Adv. 2024 Oct 2;6(24):6303–16. doi: 10.1039/d4na00541d (PMC11488687; doi:10.1039/d4na00541d)
Supplement: NA-006-D4NA00541D-s001 [file NA-006-D4NA00541D-s001.pdf]

Supplementary Information (SI) for Nanoscale Advances.
This journal is © The Royal Society of Chemistry 2024

Supporting Information

Quantum interference features and thermoelectric properties of macrocyclic-single molecules: Theoretical and modelling investigation

Sarah Hussein Halboos[a], Oday A. Al-Owaedi*[ab], Enas M. Al-Robayi[a]

[a]Department of Laser Physics, College of Science for Women, University of Babylon, Hilla 51001, Iraq.
[b]Al-Zahrawi University College, Karbala, Najaf-Karbala Street, 56001, Iraq.

*Corresponding Author: oday.alowaedi@uobabylon.edu.iq

Introduction

Studies of the electrical conductance of single molecules attached to metallic electrodes not only probe the fundamentals of quantum transport but also provide the knowledge needed to develop future molecular-scale devices and functioning circuits. [1-5] Owing to their small size (on the scale of angstroms) and the large energy gaps (on the scale of eV), transport through single molecules can remain phase coherent even at room temperature, and constructive or destructive quantum interference (QI) can be utilized to manipulate their room temperature electrical [4-6] and thermoelectrical [7,8] properties. In this work, a combination of density functional theory (DFT) [9,10] methods, a tight binding (Hückel) [11] model (TBHM) and quantum transport theory (QTT) [12-21] have been utilized to inspect the thermoelectric and electronic properties of cycloparaphenylene (CPP) molecules.

Computational Methods

All calculations in this work were carried out by the implementation of DFT in the SIESTA [22] code. It is used to obtain the optimized geometries of the structures, as shown in Figure S2. SIESTA software is an acronym derived from the Spanish Initiative for Electronic Simulations with Thousands of Atoms. The quantum transport theory (QTT) implemented in GOLLUM [13] software which is a program that computes the charge, spin and electronic contribution to the thermal transport properties of multi-terminal junctions has been utilized to calculate the electronic and thermoelectric properties of all molecular junctions in this work. All, theories and computational methods and procedures are shown in Figure S1.

The optimized geometry, ground state Hamiltonian and overlap matrix elements of each structure were self-consistently obtained using the SIESTA implementation of density functional theory (DFT). The generalized gradient approximation (GGA) of the exchange and correlation functional is used with a double-ζ polarized (DZP) basis set, a real-space grid defined with an equivalent energy cut-off of 250 Ry. The geometry optimization for each structure is performed to the forces smaller than 20 meV/Å. The mean-field Hamiltonian obtained from the converged DFT calculation was combined with GOLLUM. The transmission coefficient T(E) for electrons of energy E (passing from the source over molecule to the drain) is calculated via the relation:

$$T(E) = T_r\left\{\Gamma_R(E)G^R(E)\Gamma_L(E)G^{R\dagger}(E)\right\} \text{..............} (1)$$

In this expression,

$$\Gamma_{L,R}(E) = i\left(\Sigma_{L,R}(E) - \Sigma_{L,R}^{\dagger}(E)\right) \text{..........................} (2)$$

$\Gamma_{L,R}$ describes the level broadening due to the coupling between left (L) and right (R) electrodes and the central scattering region, $\Sigma_{L,R}(E)$ are the retarded self-energies associated with this coupling.

$$G^R = (ES - H - \Sigma_L - \Sigma_R)^{-1} \text{ .... (3)}$$

G^R is the retarded Green's function, where H is the Hamiltonian and S is the overlap matrix (both of them are obtained from SIESTA). The transport properties is then calculated using the Landauer formula:

$$G(E_F T) = G_\circ \int_{-\infty}^{\infty} dE\, T(E)\left[-\partial f(E,T,E_F)/\partial E \right] \text{ .... (4), where}$$

$$f = \left[e^{(E - E_F)} \Big/ k_B T + 1 \right]^{-1} \text{ .... (5)}$$

is the Fermi-Dirac probability distribution function, T is the temperature, E_F is the Fermi energy, $G_\circ = (2e^2)/h$ is the conductance quantum, e is electron charge and h is the Planck's constant. DFT can give inaccurate value for the Fermi energy that the calculated conductance are obtained for a range of Fermi energies. [23] The thermopower or Seebeck coefficient (*S*) is defined as the difference of electrochemical potential per unit temperature difference developing across an electrically isolated sample exposed to a temperature gradient. The Seebeck coefficients and power factors is also informative. Provided the transmission function T(E), can be approximated by a straight line on the scale of K_{BT}, the Seebeck coefficient is given by:

$$S \approx -L|e|T\left(\frac{dln\, T(E)}{dE}\right)_{E = E_F} \text{ .... (6)}$$

Where L is the Lorenz number $L = \left(\frac{k_B}{e}\right)^2 \frac{\pi^2}{3} = 2.44 \times 10^{-8}$ WΩK^{-2} In other words, *S* is proportional to the negative of the slope of lnT(E), evaluated at the Fermi energy.

The power factor is the ratio of the real power absorbed by the load to the apparent power flowing in the circuit. Real power is the average of the instantaneous product of voltage and current and represents the capacity of the electricity for performing work. From the Seebeck coefficient, the power factor was calculated as given in equation (7)

$P = GS^2 T$ ……………………………… (7)

where T is the temperature T = 300 K, G is the electrical conductance and *S* is the thermopower. In conventional devices the maximum efficiency of either heat transfer or current generation is proportional to the dimensionless thermoelectric figure of merit. The common measure for thermoelectric efficiency is given by the figure of merit, which is given by: [24]

$ZT = \frac{GS^2}{k_{el} + k_{ph}} T$ ………………………………. (8), where G is the electrical conductance, *S* is the thermopower, k_{el} is the electron thermal conductance, $k_{ph}$is the phonon thermal conductance. The figure of merit is determined from the thermoelectric transport coefficients in equations 6, 9-10, and 12 in the linear response regime. [25-27]

$$G = \frac{2e^2}{h} k_0$$ ……………………………………………… (9)

$$k_{el} = \frac{2}{hT}\left(K_2 - \frac{K_1^2}{K_0}\right)$$ ………………………………. (10)

In the expressions $e = |e|$ is the absolute value of the electron charge, h is the Planck constant, and $T = (T_L + T_R)/2$ is the average junction temperature. The coefficients in 9 and 10 are defined as:

$$k_n = \int dE\, T_{el}(E)\left(-\frac{\partial f(E)}{\partial E}\right)(E-\mu)^n \quad \text{.............................. (11)}$$

Where $T_{ph}(E)$ is the electron transmission, and the chemical potential $\mu \approx E_F$ is approximately given by the Fermi energy E_F of the Au electrodes.

The corresponding thermal conductance due to the phonons is given in linear response by:

$$k_{ph} = \frac{1}{h}\int_0^{\infty} dE\, ET_{ph}(E)\frac{\partial n(E,T)}{\partial T} \quad \text{...................................... (12)}$$

Where $T_{ph}(E)$ is the phonon transmission and $n(E,T) = \{\exp(E/_{kB}T) - 1\}^{-1}$ is the Bose function, characterizing the phonon reservoirs in the left and right electrodes.

Hence, an upper bound for ZT in the limit of vanishing phonon thermal transport $\kappa_{ph} \rightarrow 0$ is given by the purely electronic contribution [25] as

$$Z_{el}T = \frac{S^2G}{k_{el}}T = \frac{S^2}{L} \quad \text{............................... (13)}$$

Hence, the Lorenz number is $L = \kappa_{el}/GT$. With $Z_{el}T$, and depending on above the figure of merit is presented in a slightly different form as:

$$ZT = \frac{Z_{el}T}{1 + {k_{ph}}/{k_{el}}} \quad \text{.................................... (14)}$$

The initial optimization of gas phase molecules and isosurfaces calculations were carried out at the B3LYP level of theory [28] with 6-31G** basis set [29,30] using density functional theory (DFT) and time-dependent (TD-DFT) 31 respectively. B3LYP is one of the most accurate and popular

DFT functional, and gives good results. [28-30] It is a so-called hybrid functional and is usually expressed in the following form:

$$F^{B3LYP}_{XC} = 1 - aF^{Slater}_{X} + aF^{HF}_{X} + bF^{B88}_{X} + cF^{LYP}_{C} + 1 - cF^{VWN}_{C} \tag{15}$$

where F_X^{Slater} refers to the Slater exchange, F_X^{HF} is the Hartree–Fock exchange, F_X^{B88} is the Becke's exchange functional. F_C^{LYP} is the correlation functional of Lee, Yang, and Parr, and F_C^{VWN} is the correlation functional of Vosko, Wilk, and Nusair. The coefficients are a = 0.20, b = 0.72, and c = 0.81, which were adapted from another hybrid functional, B3PW91. The values of the coefficients were originally determined empirically by a linear least-squares fit to 116 experimentally determined energies.

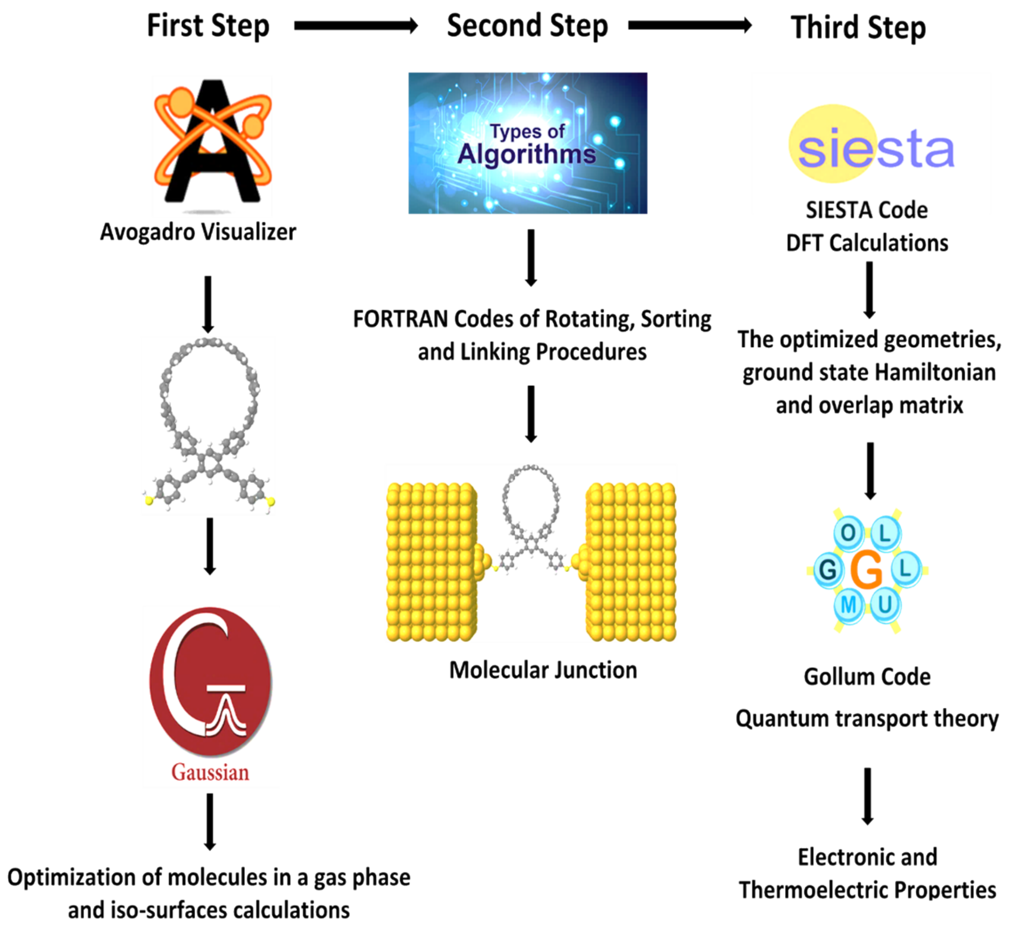

Figure S1. Computational methods and steps.

All molecules in a gas phase has been designed using Avogadro [31] visualizer, then the ground-state energy optimization of molecules and iso-surfaces calculations achieved using Gaussian [32] software at the B3LYP level of theory [28] with 6-31G** basis set. [29,30] The second step involves the rotation, sorting and linking the molecules to the gold electrodes to obtain the theoretical models of molecular junctions (see Figure S2), using a set of FORTRAN algorithms. After that the molecular junctions have been optimized using SIESTA. [22] The Hamiltonian and overlap matrix were then fed to the Gollum [13,33] code, which calculating the electronic and thermoelectric properties of all molecular junctions.

Theoretical models

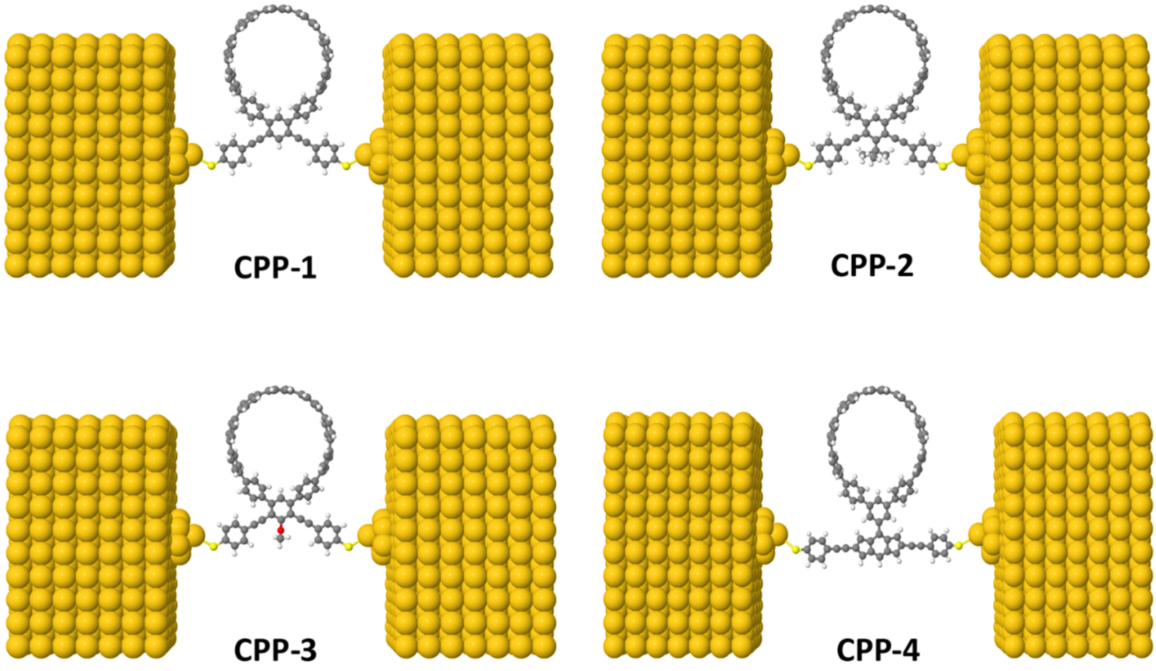

Figure S2. Theoretical models of optimized molecular junctions.

The theoretical models of all molecular configurations consist of optimized molecules attached two (111)-directed gold electrodes involving small 6-atom pyramidal gold leads, and

each electrode constructed of eight layers of (111)-oriented bulk gold with each layer consisting of 6 × 6 atoms, and a layer spacing of 0.235 nm were employed to create the molecular junctions. These layers were then further repeated to yield infinitely long current carrying gold electrodes. From these model junctions the electronic and thermoelectric properties were calculated using the GOLLUM code.

The Negative Differential Resistance (NDR) of CPP Molecules

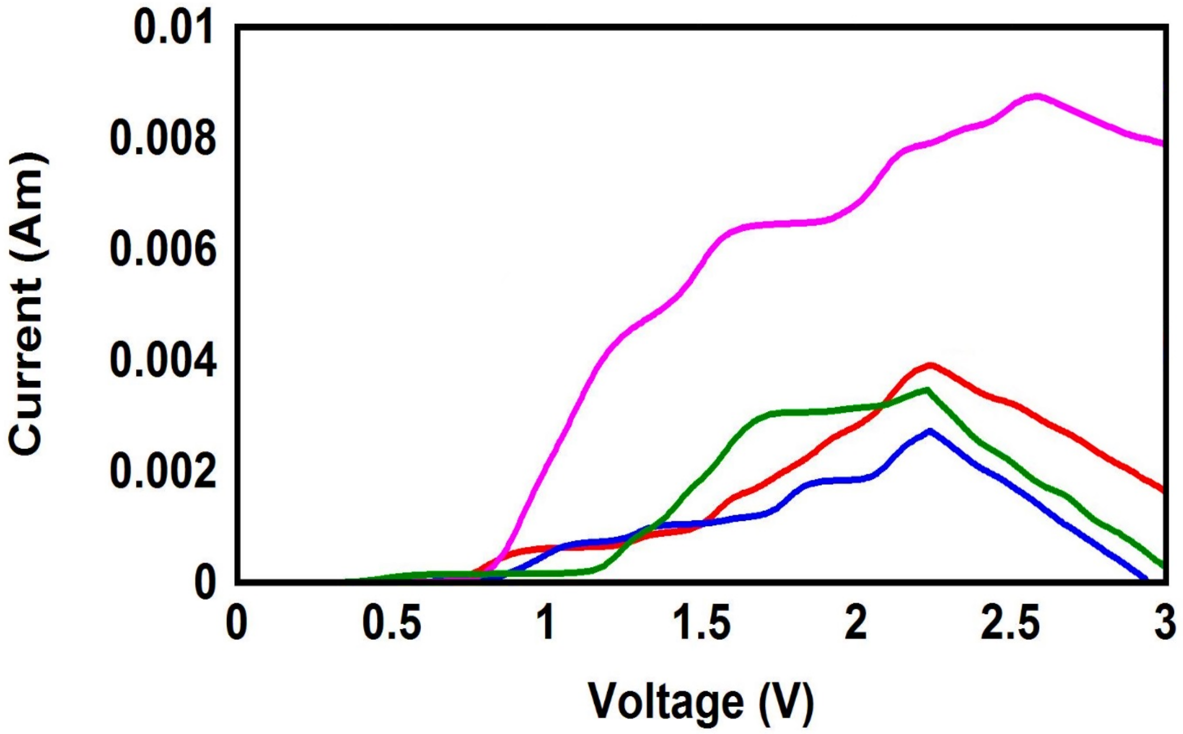

Figure S3. Current-voltage characteristics of all molecular junctions.

The current is increasing obviously by the increasing bias voltage. When the bias voltage is further increased to a certain range [2, 2.5] V, the current decreases and the negative differential resistance (NDR) appears [34], as shown in Figure S3. These results are consistent with the results of reference 33.

Seebeck Coefficient of CPP-1 Molecule

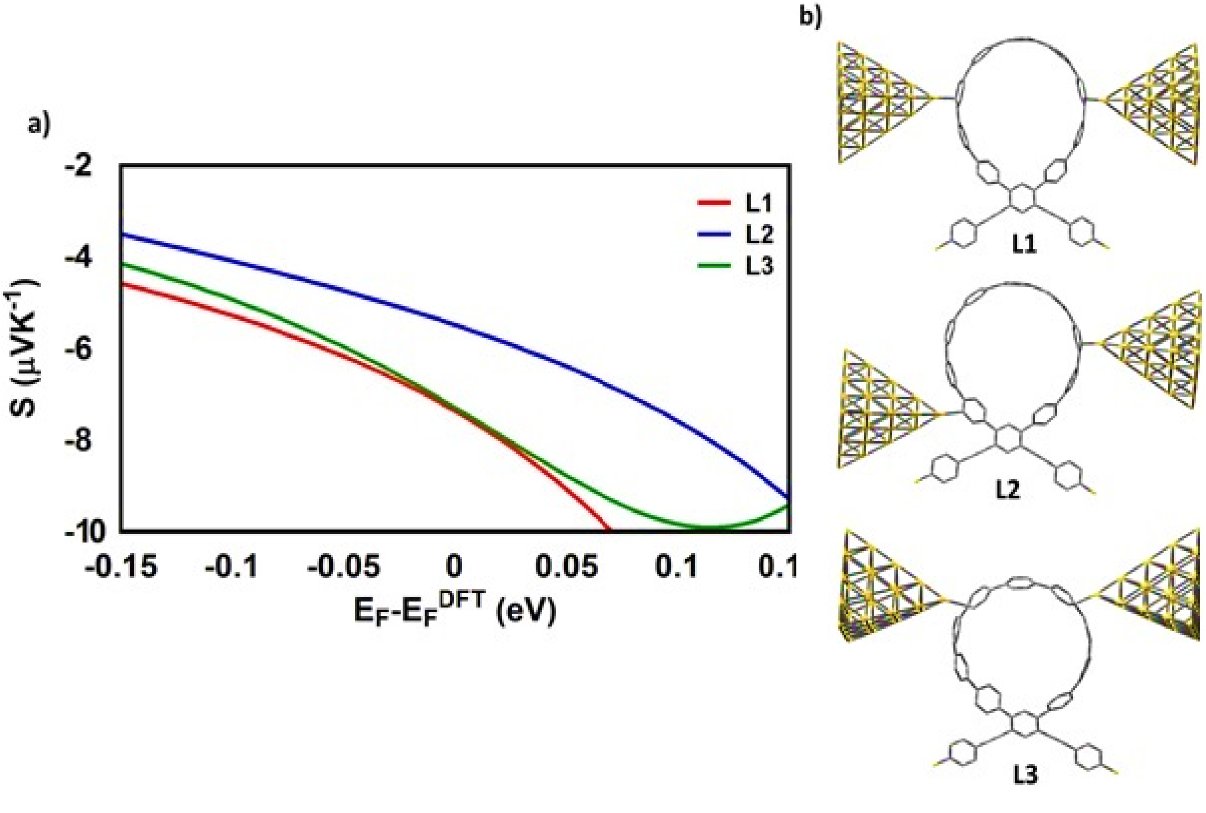

Figure S4. a) Seebeck coefficient (S) as a function of Fermi energy of CPP-1 molecular junction as an example; b) Theoretical models of optimized molecular junctions in different junction

formation probabilities. L1 is the meta-meta connection; L2 is the meta-ortho connection; L3 is the para-para connection.

The theoretical models shown in Figure S4, have simulated the probably contacts of gold electrodes to carbon atoms in the circular wheel of structure CPP-1 with various connections. The L1 model involves the formation of the molecular junction via a meta-meta connection. In contrast, the L3 model shows the structure of molecular junction with a para-para connection. The third model (L2) exhibits a mixed connection consist of meta and ortho positions. The values of Seebeck coefficient (*S*) of all models are quiet small ranging from -7.4 to -5.5 μVK^{-1} and the sign of *S* is negative.

Tight-Binding Hückel Model

The neglect of tight-binding Hückel model (TBHM) of the interactions between electrons is considered a major defect, but it remains one of the widely used methods to visualize and understand the electronic properties of molecular junctions. [35] One of the drawbacks of this kind of computational methods is the produced energy levels are diminished by a few eV in comparison with the accurate values relative to a vacuum, but the energy variances are usually appropriate to compare with DFT calculations. Therefore, this method is considered a powerful tool to award reasonable and precise results that could figure out the fundamental physics and resolve the problems. In seeking to understand the transport behaviour of molecules and the relative effect of different transport connections (para or meta), and the influnce of pendant groups such as 2-methylpropane and methoxy, a minimal tight-binding (Hückel) model (TBHM) has been constructed, as shown in Figure S5. The simplest tight-

binding Hamiltonian of the parents is obtained by assigning a site energy ε to each diagonal and a nearest neighbor hopping integral γ between neighbouring sites, i.e., $H_{ii} = \varepsilon$ and $H_{ij} = \gamma$ if i, j are the nearest neighbours.

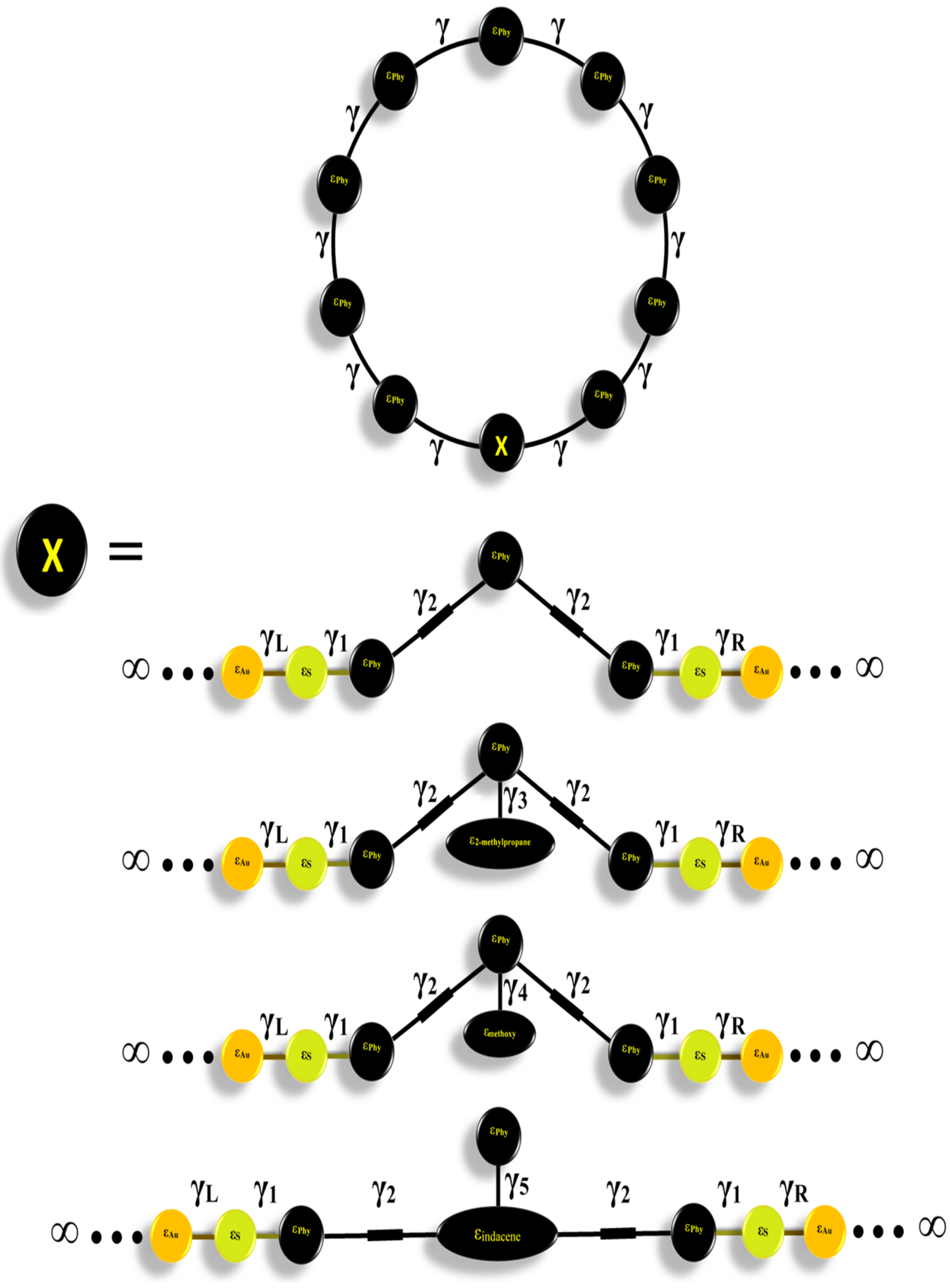

Figure S5. A minimal tight-binding (Hückel) representation of CPP molecules with different intramolecular coupling elements, γ, and different onsite energies ε.

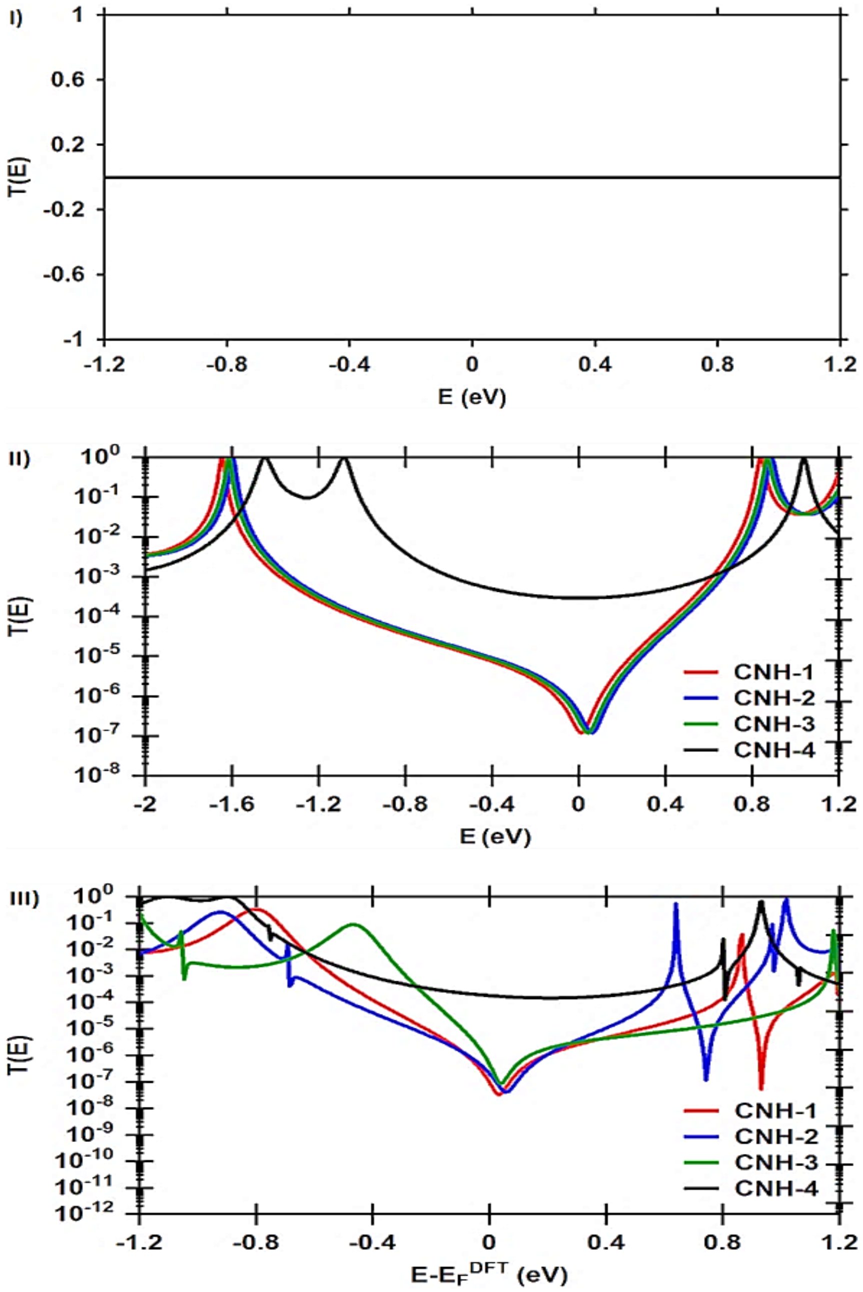

Figure S6. **I**) Transmission coefficients as a function of electrons energy for CPP molecules, where model **I** is the results of TBHM with all on-site energies equal 0 eV, and all coupling integrals equal -1 eV. **II**) T(E) for all CPP molecules, where model **II** is the results of TBHM with coupling integrals (γ = γ1 = γL = γR = 0.5 and γ2 = 0.3 eV) for all models, and γ3 = 2.2, γ4 = 0.5 and γ_5 = 0.9 eV), and the on-site energies (ε_{phy} = 0.5, $\varepsilon_{2\text{-methylpropane}}$ = 0.7, $\varepsilon_{methoxy}$ = 0.7 and $\varepsilon_{indacene}$ = 1.1 eV). **III**) DFT-Transmission coefficients as a function of Fermi energies for CPP molecules.

At the beginning, all sites are treated as the same and set the on-site energies to 0 eV and coupling integrals to -1 eV, the resulting transmissions are disagree with the DFT results, as shown in Figure S6I. However, by adjusting the coupling integrals ($\gamma = \gamma_1 = \gamma_L = \gamma_R$ = 0.5 and γ_2

= 0.3 eV) for all models, and γ_3 = 2.2, γ_4 = 0.5 and γ_5 = 0.9 eV), as well as, by changing the on-site energies (ε_{phy} = 0.5, $\varepsilon_{2\text{-methylpropane}}$ = 0.7, $\varepsilon_{methoxy}$ = 0.7 and $\varepsilon_{indacene}$ = 1.1 eV), the TBHM results are reproduced, and they are consistent (in terms of transmission value) with DFT results as shown in figures S6II,III.

References

1 J. Chen, M. A. Reed, A. M. Rawlett, J. M. Tour, *Science* 1999, **286**, 1550-1552.
2 M. A. Reed, C. Zhou, C. J. Muller, T. P. Burgin, J. M. Tour, *Science* 1997, **278**, 252-254.
3 S. Bock, O. A. Al-Owaedi, S. G. Eaves, D. C. Milan, M. Lemmer, B. W. Skelton, H. M. Osorio, R. J. Nichols, S. J. Higgins, P. Cea, N. J. Long, T. Albrecht, S. Martin, C. J. Lambert, P. J. Low, *Chem. Eur. J.* 2017, **23**, 2133-2143.
4 C. Jia, X. Guo, *Chem. Soc. Rev.* 2013, **42**, 5642-5660.
5 B. A. A. Al-Mammory, O. A. Al-Owaedi, E. M. Al-Robayi, *J. Phys. Conf. Ser.* 2021, **1818**, 012095.
6 S. Ballmann, R. Härtle, P. B. Coto, M. Elbing, M. Mayor, M. R. Bryce, M. Thoss, B. H. Weber, *Phys. Rev. Lett.* 2012, **109**, 056801.
7 Eichler, J. C. A., J. M. G. Ceballos, R. Rurali, A. Bachtold, *Nat. Nanotechnol.* 2012, **7**, 301-304.
8 C. M. Finch, V. M. Garcia-Suarez, C. J. Lambert, *Phys. Rev. B,* 2009, **79**, 033405.
9 J. P. Perdew, K. Burke, M. Ernzerhof, *Phys. Rev. Lett.* 1996, **77**, 3865.
10 P. J. Stephens, F. J. Devlin, C. F. Chabalowski, M. J. Frisch, *J. Phys. Chem.* 1994, **98**, 11623-11627.
11 E. Estrada, *Proc. R. Soc. A,* 2017, **474**, 0721.
12 J. P. Perdew, Y. Wang, *Phys. Rev. B,* 1992, **45**, 13244.
13 J. Ferrer, C. J. Lambert, V. M. García-Suárez, D. Zs Manrique, D. Visontai, L. Oroszlany, R. Rodríguez-Ferradás, I. Grace, S. W. D. Bailey, K. Gillemot, H. Sadeghi, L. A. Algharagholy, *New J. Phys.* 2014, **16**, 93029.
14 O. A. Al-Owaedi, T. T. Khalil, S. A. Karim, E. Al-Bermany, D. N. Taha, *Systematic Reviews in Pharmacy* 2020, **11**, 110-115.
15 J.C. Judd, A. S. Nizovtsev, R. Plougmann, D. V. Kondratuk, H. L. Anderson, E. Besley, A. Saywell, *Phys. Rev. Lett.* 2020, **125**, 206803.
16 Z. Chen, J-R. Deng, S. Hou, X. Bian, J. L. Swett, Q. Wu, J. Baugh, L. Bogani, G. A. D. Briggs, J. A. Mol, C. J. Lambert, H. L. Anderson, J. O. Thomas, *J. Am. Chem. Soc.* 2023, **145**, 15265-15274.
17 S. Richert, J. Cremers, I. Kuprov, M. D. Peeks, H. L. Anderson, C.R. Timmel, *Nat. Commun.* 2017, **8**, 14842.
18 B. Limburg, J. O. Thomas, G. Holloway, H. Sadeghi, S. Sangtarash, J. Cremers, A. Narita, K. Müllen, C. J. Lambert, G. A. D. Briggs, J. Mol, H. L. Anderson, *Adv. Func. Mat.* 2018, **28**, 1803629.
19 G. Sedghi, L. J. Esdaile, H. L. Anderson, V. M. García-Suárez, C.J. Lambert, S. Martin, D. Bethell, S. J. Higgins, R. J. Nichols, *Nature Nanotechnol.* 2011, **6**, 517-523.
20 N. Algethami, H. Sadeghi, S. Sangtarash, C. J. Lambert, *Nano Lett.* 2018, **18**, 4482-4486.

21 E. Leary, B. Limburg, S. Sangtarash, A. Alanazy, I. Grace, K. Swada, L. J. Esdaile, M. M. Noori, T. González, G. Rubio-Bollinger, H. Sadeghi, N. Agrait, A. Hodgson, S. J. Higgins, C. J. Lambert, H. L. Anderson, R. J. Nichols, *J. Am. Chem. Soc.* 2018, **140**, 12877-12883.
22 J. P. Perdew, K. Burke, M. Ernzerhof, *Phys. Rev. Lett.* 1996, **77**, 3865.
23 C. J. Lambert, *Chem. Soc. Rev.* 2015, **44**, 875-888.
24 C. J. Judd, A. S. Nizovtsev, R. Plougmann, D. V. Kondratuk, H. L. Anderson, E. Besley, A. Saywell. *Phys. Rev. Lett.* 2020, **125**, 206803.
25 U. Sivan, Y. Imry, *Phys. Rev. B,* 1986, **33**, 551.
26 K. Esfarjani, M. Zebarjadi, Y. Kawazoe, *Phys. Rev. B,* 2006, **73**, 085406.
27 K. H. Müller, *J. Chem. Phys.* 2008, **129**, 044708.
28 A. D. J. Becke, *Chem. Phys.* 1993, **98**, 5648-5652.
29 G. A. Petersson, A. Bennett, T. G. Tensfeldt, M. A. Al-Laham, W. A. Shirley, J. Mantzaris, *J. Chem. Phys.* 1988, **89**, 2193-2198.
30 G. A. Petersson, M. A. Al-Laham, *J. Chem. Phys.* 1991, **94**, 6081-6090.
31 H. D. Marcus, C. E. Donald, L. C. David, V., Tim, Z. Eva, H. R. Geoffrey, *J. Chem. Inform.* 2012, **4**, 1-17.
32 H. B. Schlegel, J. S. Binkley, J. A. Pople, *J. Chem. Phys.* 1984, **80**, 1976-1981.
33 H. Ozawa, M. Baghernejad, O. A. Al-Owaedi, V. Kaliginedi, T. Nagashima, J. Ferrer, T. Wandlowski, V. M. García-Suárez, P. Broekmann, C. J. Lambert, M. Haga, *Chem. Eur. J.* 2016, **22**, 12732-12740.
34 H. Lizhi, G. Yandong, Y. Xiaohong, Z. Hongli, Z. Jie, *Phys. Lett. A* 2017, **381**, 2107-2111.
35 K. Walczak, E. S. Lyshevski, *centr. Eur. J. Phys.* 2005, **3**, 555-563.
